# An ethics analysis of the rationale for publicly funded plastic surgery

**DOI:** 10.1186/s12910-020-00539-6

**Published:** 2020-10-02

**Authors:** Lars Sandman, Emma Hansson

**Affiliations:** 1 National Centre for Priorities in Health, Department of Health, Medicine and Caring Sciences, Linköping University, S-581 83 Linköping, Sweden; 2 Västra Götaland Region, Sweden; 3 Borås University, Borås, Sweden; 4 Department of Plastic and Reconstructive Surgery, Sahlgrenska University Hospital, Gröna Stråket 8, SE-413 45 Gothenburg, Gröna Stråket 8, SE-413 45 Gothenburg, Sweden; 5 The Sahlgrenska Academy, Gothenburg University, Gothenburg, Sweden

**Keywords:** Plastic surgery, Esthetic surgery, Rationing, Prioritizing, Normality, Functional condition, Psychosocial condition, Etiology, Healthcare need, Patient experience

## Abstract

**Background:**

Healthcare systems are increasingly struggling with resource constraints, given demographic changes, technological development, and citizen expectations. The aim of this article is to normatively analyze different suggestions regarding how publicly financed plastic surgery should be delineated in order to identify a well-considered, normative rationale. The scope of the article is to discuss general principles and not define specific conditions or domains of plastic surgery that should be treated within the publicly financed system.

**Methods:**

This analysis uses a reflective equilibrium approach, according to which considered normative judgements in one area should be logically and argumentatively coherent with considered normative judgements and background theories at large within a system.

**Results and conclusions:**

In exploring functional versus non-function conditions, we argue that it is difficult to find a principled reason for an *absolute* priority of functional conditions over non-functional conditions. Nevertheless, functional conditions are relatively easier to establish objectively, and surgical intervention has a clear causal effect on treating a functional condition.

Considering non-functional conditions that require plastic surgery [i.e., those related to appearance or symptomatic conditions (not affecting function)], we argue that the patient needs to experience some degree of suffering (and not only a preference for plastic surgery), which must be ‘validated’ in some form by the healthcare system.

This validation is required for both functional and non-functional conditions. Functional conditions are validated by distinguishing between statistically normal and abnormal functioning. Similarly, for non-functional conditions, statistical normality represents a potential method for distinguishing between what should and should not be publicly funded. However, we acknowledge that such a concept requires further development.

## Background

Healthcare systems are increasingly struggling with resource constraints, given demographic changes, technological development, and citizen expectations. In publicly funded welfare-type healthcare systems (i.e., with a strong emphasis on equal access to healthcare), this implies a recurrent re-evaluation of what should be reimbursed and what should be rationed. Decisions on reimbursement can be made at different levels of such systems, including national (e.g., France and The Netherlands) or regional (the Swedish system) [1] However, regardless of where decisions are made, the strong norm of equality in such systems will require standardization regarding what is reimbursed. Determination of this standard depends on the size of the healthcare budget, which introduces ‘borderline’ effects (i.e., types of healthcare interventions found both ‘inside’ and ‘outside’ of the budget). One such area is plastic surgery, whereas another might be assisted reproduction.

Some areas of plastic surgery, such as reconstructions after trauma, burns, cancer, and congenital malformations, should indisputably be part of a publicly funded healthcare system. However, other interventions are more disputed, and there is ongoing discussion regarding reimbursement and rationing in plastic surgery [2–4]. Even under these conditions, the threshold concerning what should be offered in a publicly funded system remains to be determined. For example, how many cosmetic corrections should patients be offered, and what type and degree of defects should patients have to accept? Analyses of guidelines from England [5, 6] describe the existence of a postcode lottery concerning whether or not plastic surgery is offered as part of public healthcare, indicating the difficulty to agree on a common, evidence-based, and clinically applicable sorting method [4]. Moreover, there is increasing pressure from citizens to offer plastic surgery in the publicly funded healthcare system based on the perceived changes in need and expectations according to influences from the media [7, 8], Internet [9], and social factors [10].

The aim of this article is to normatively analyze different suggestions regarding how publicly financed plastic surgery should be delineated in order to identify a well-considered, normative rationale. The scope of the article is to discuss general principles rather than define specific conditions or domains of plastic surgery that should be treated within a publicly financed system.

In plastic surgery, there have been attempts to distinguish between what should be reimbursed or rationed by differentiating between functional and non-functional conditions, reconstructive and esthetic surgery, and normal and abnormal status, as well as by referring to different condition-specific etiologies [4]. The American Medical Association (AMA) uses the following definitions of ‘esthetic’ and ‘reconstructive’ surgery:” Cosmetic surgery is performed to reshape normal structures of the body in order to improve the patient’s appearance and self-esteem. Reconstructive surgery is performed on abnormal structures of the body caused by congenital defects, developmental abnormalities, trauma, infection, tumors, or disease. It is generally performed to improve function but may also be done to approximate a normal appearance” [11]. Here, we will show that this does not provide adequate guidance for priority setting. Additionally, there is a recurrent discussion concerning the role of subjective perceptions or preferences by the patient in deciding whether plastic surgery should be reimbursed [4, 6]. We have not found any in-depth normative analysis of these different suggestions and their internal relationship.

Our analysis has the following outline. First, we explore the distinction between functional and non-functional conditions. We then focus on non-functional conditions and explore the role of patient preferences, subjective experiences, and normality relative to non-functional conditions. In exploring normality, we evaluate whether different etiologies of a non-functional condition should make a difference in whether the problem can be assessed as norm. The article ends with our central conclusions.

## Methods

This analysis uses a reflective equilibrium approach, according to which considered normative judgements in one area should be coherent with considered normative judgements and background theories within a system at large [12, 13]. Specifically, this requires identification of general principles for distribution of scarce resources in the healthcare system and how those principles are applied when distributing resources in other parts of the healthcare system. Additionally, it requires analysis of whether this is consistent with suggestions regarding how to delineate public funding of plastic surgery identified in the literature. Moreover, these principles and the normative judgments they imply, should also be consistent with the empirical environment in which they are applied, in this case empirical data about how different relevant conditions affect patients, how plastic surgery can treat those conditions and the resulting effects on patients etc.

Coherence implies logical or argumentative coherence, hence applying a reflective equilibrium methodology means showing through logically consistent argumentation whether suggestions can be supported or not. Coherence does not necessarily imply agreement between different stakeholders or individuals, any such disagreement has to be supported by pointing to flaws in the analysis or argumentation. In this article we will analyze a number of considered judgements (see below), explicitly or implicitly found in the literature or in practice against a set of normative principles for distributing scarce resourses [14].

In this study, we make the following assumptions about normative principles for guiding resource distribution in a publicly financed, welfare-type healthcare system: 1) only healthcare needs should be treated within publicly funded healthcare systems (what we will call the principle of exclusivity of needs or PEN for short); 2) the greater the size of the healthcare need, the more warranted it is to receive funding ceteris paribus *(*what we will call the principle of size of needs or PSE for short*)*; 3) if the treatment of similar problems is funded elsewhere in the healthcare system, this requires a special reason not to fund it within the context of plastic surgery and vice versa (what we will call the principle of formal equality or PFE for short) [15]. The analysis is undertaken relative to publicly financed, welfare-type, and needs-based healthcare systems, such as the British National Health System or the Scandinavian healthcare systems. Because the focus of the analysis is on distribution of scarce resources (i.e., distributive justice), other relevant clinical ethics considerations related to principles of beneficence, non-maleficence, and autonomy will only be mentioned in passing if relevant to the focus on justice [15].

In the article we will explore whether the following considered judgements are consistent with PEN, PSE and PFE:

- CJ1: Functional conditions should be prioritized to public funding before non-functional conditions.
- CJ2: If patients have a preference for plastic surgery, it should be publicly funded.
- CJ3: If patients report suffering that can be reduced with plastic surgery it should be publicly funded.
- CJ4: If patients have validated suffering that can be reduced with plastic surgery it should be publicly funded.
- CJ5: If patient have validated suffering that can be reduced with plastic surgery and their condition is outside the range of what is statistically normal, it should be publicly funded.

## Results and discussion

### Healthcare need and the distinction between functional and non-functional conditions

In publicly financed, welfare-type healthcare systems, the concept of healthcare need is essential to determining whether interventions are reimbursed [16]. Normally, status as a healthcare need is a necessary (but insufficient) criterion for reimbursement. Recent analytical developments suggest that a healthcare need should be understood as a three-part concept with the following structure [17, 18]:

- P has a healthcare need for intervention Y if P benefits by moving from Z_current level_ toward Z_reference level_ through Y.

P is a person or patient (whatever concept is preferred), Y is an intervention or type of intervention used within the healthcare system, and Z labels the value objective of the healthcare system. Because the type of interventions used in plastic surgery are the same as those in any surgical specialty, we assume there is no quarrel regarding Y. Considering Z, there have been numerous attempts to define the healthcare objective, which represents a consistently evolving effort [19]. However, it is notoriously difficult to determine a generally acceptable definition; therefore, we will use a more pragmatic or ‘casuistic’ approach in this article by assuming that some broad version of health is the healthcare objective. Generally, we can understand health in terms of a quality of life dimension (what is the quality of a person’s life at each point in time) and a life-length dimension (how long is the life of the person). In this context we will (almost) exclusively focus on the quality of life dimension of health, where we broadly distinguish between the *physical functioning* of a person (how the body works) and a person’s experienced quality of life (in this case focused on the negative side of this, *suffering*). This is a simplification, especially in how we use the concept of suffering, but still we believe that this is sufficient to argue our point in this article [19]. When analyzing plastic surgery, we evaluate how comparable problems are handled elsewhere in the healthcare system, with the intention of arriving at consistency and identifying borderline cases. It is generally assumed that the objective of healthcare is not necessarily ideal or optimal health but rather acceptable health, given the health level of the society in question when operating under scarce resources. Therefore, it is assumed that Z_current level_ < Z_reference level_, implying that there could be levels of health above the reference level, and that different conditions can be equivalent to the reference level of health. Looking at that proposed definition, we find that there is both, what we might call a severity component of healthcare need, i.e. how far the patient is from the Z_reference level_ in the current situation and to what extent the healthcare intervention Y can bring her closer to Z_reference level_, what we might call the effectiveness of treatment. Hence, our principle PSE, will have two aspects to consider, a severity and an effectiveness aspect.

### Functional and non-functional conditions

Let us start by analyzing CJ1 (that functional conditions should be prioritized before non-fuctional conditions to public funding). The distinction between a functional and non-functional condition is common in plastic surgery, voicing the distinction between esthetic and reconstructive surgery from the AMA [11]. This distinction is far from clear, but in this article we will use it in the following way (drawing on the above conceptualization of health). A functional condition is one involving impairment of a *physical* function, where impairment will imply functioning outside of what is statistically normal for the population the patient belongs to (see below). A non-functional condition, by distinction, does not involve impairment of a physical function. Generally, in this article, non-functional conditions will imply a condition related to the appearance of the person. In some cases, as will be shown below, non-functional conditions might indirectly result in physical dysfunction, e.g. when patients abstain from healthcare interventions since it would affect their appearance negatively or when it results in excessive weight loss or training. However, at this point we hope the distinction between physical dysfunction and problems related to appearance is clear enough for the analysis. Following this, it seems CJ1 aligns with PEN, i.e. functional conditions are indeed healthcare needs.

The assumption is that a physical functional condition lowers health to a greater degree and is better suited for surgical treatment than non-functional conditions resulting from the experienced appearance of the patient [4, 20]. In other words that CJ1 aligns with PSE. Therefore, a functional condition warrants publicly funded plastic surgery to a greater extent than a non-functional condition. For example, qualifying for abdominoplasty requires more than the presence of a significant amount of excess skin and the associated suffering. The problem needs to exist in combination with a functional condition, such as eczema, infections, or micturition difficulties [3, 21]. Moreover, healthcare systems might perform breast reductions to alleviate back symptoms, although normally not breast augmentations, even when small breasts might cause some degree of suffering [3, 4, 22]. Experiencing a functional condition can obviously give rise to suffering, but it can also be a problem, despite patient experience (e.g., increased risk of premature death, a risk of future suffering). Additionally, the condition might introduce an objectively observable functional limitation, in which case it might be easier to objectively establish a healthcare need. For example, reducing breast size will relieve back strain, or abdominoplasty will remove the environment where eczema and infections thrive. Such reasoning makes it easier for both the patient and surgeon to classify the operation as medically warranted and not purely an esthetic intervention [23]. To define healthcare needs in terms of objectively quantifiable indications also provides the patient and surgeon with a feeling that the procedures are legitimate and offered in a consistent way according to the requirements of equity [23]. However, such a statement needs qualification.

First, we need a standard for when a functional variation qualifies as a functional condition. Generally, finding such a standard requires identifying a range of variations outside of which the risk of a certain negative outcome is high enough. Another way to express this is in terms of statistical normality according to Boorse [24]. If an organ is functioning inside a range of what is statistically normal in a population, there is no problem; however, if there is a malfunction in relation to this range, there is a problem. Therefore, we need to define this range in order to identify when a healthcare intervention is warranted. Breast reduction to allow a patient to live with slightly smaller breasts that decrease back strain might not represent a large enough functional problem. Similarly, harboring excess skin with a small risk of eczema or infection might not be a large enough functional problem. Nevertheless, if there is a functional condition, but the patient does not experience or value the consequences of this as a problem, there is no healthcare need. Note that this does not exclusively concern what is experienced as a problem in the present. We normally do not experience risks, but we might still value the potential outcome of a risk as a problem. However, if there is a functional condition and the patient does not find either the present or the potential future outcomes as problematic (even in principle), it would be strange to claim the existence of a healthcare need. Plastic surgery is needed to avoid premature death associated with (for example) large burn wounds and extensive tissue defects after removal of advanced tumors, such as head-and-neck, colorectal, and gynecologic cancers, and after trauma. In these cases, avoiding premature death is important to the extent that a patient wants to continue living (under the given circumstances). Eczema, infections, and back pain give rise to suffering. In some instances, functional conditions might lead to worse problems requiring more extensive interventions if not addressed. For example, failure to perform surgery on a scar contracture causing impeded physical function, such as restricted arm movement, could potentially eliminate the possibility to rehabilitate joints, thereby negatively affecting QoL. Therefore, patient experience and values need to be accounted for while assessing the existence of a healthcare need. This illustrates the difficulty in drawing a clear distinction between functional and other conditions, given their movement on the same scale. Hence, in effect, it is not obvious that functional conditions always better align with PEN than non-functional conditions, and aspects central to non-functional conditions are also part and parcel of functional conditions.

#### Are functional conditions more serious than non-functional conditions?

In some cases, functional conditions are obviously highly serious, as they can potentially result in unwanted premature death, and a high level of suffering. In such cases, reimbursement of plastic surgery is never an issue.

The risk of non-functional conditions resulting in premature death, or extensive suffering, should however not be underestimated. Some patients with human immunodeficiency virus (HIV) stop taking their anti-retroviral drugs and risk their lives in order to avoid the esthetic implications of the medication [25]. Moreover, other patients commit suicide due to suffering following perceived appearance conditions [26]. Empirical data suggest that esthetic conditions or an altered body image are related to psychosocial or even psychiatric conditions giving rise to suffering, and that people do suffer from experienced esthetic abnormalities and imperfections that can impede daily life [27–31]. A previous study compared patients seeking plastic surgery in the public healthcare system for functional reasons with those seeking care for esthetic reasons, finding that those seeking care for purely esthetic reasons were more distressed than population norms. Moreover, the esthetic group displayed worse social function than healthy people, whereas the functional group exhibited social function comparable to that of healthy people [28]. Furthermore, it was suggested that people receiving certain types of esthetic plastic surgery more frequently experience mental health conditions, and suffering in terms of anxiety and depression, as compared with the general population [31, 32]. These findings suggest that it is not obvious that functional conditions are always more severe than non-functional conditions and so CJ1 does not necessarily align with PSE as to the severity aspect.

#### Is plastic surgery a more effective treatment for functional conditions than for non-functional conditions?

Plastic surgery has a more directly measurable impact on functional conditions than non-functional conditions. For example, reductions in breast size reduce back strain according to the laws of physics, and if excess skin is removed, this hinders the environment for bacterial infection. The effect of plastic surgery on non-functional conditions is more indirect and, therefore, more difficult to quantify. Numerous studies show that esthetic plastic surgery improves QoL (or reduces suffering in our terminology) [33], although there is little evidence of long-term effects on psychosocial functioning and QoL and, by extension, the true benefit of the treatment [33–35]. Therefore, it remains unclear whether non-functional conditions related to appearance are best treated with plastic surgery or some other health intervention. Moreover, the relationship between suffering and plastic surgery is complex. Studies revealed that patients with mild-to-moderate distress might benefit more from surgery than those with a higher level of distress [36, 37]. In some cases, a high level of distress due to appearance conditions could be an indication of body dysmorphic disorder, in which case surgery is contra-indicated [38]. Therefore, it is not necessarily true that the patient with the most prominent experienced pre-operative dysfunction should be treated with plastic surgery. Similarly, there is no connection between psychosocial conditions and the severity of disfigurement [30]. The previous study comparing patients seeking plastic surgery for functional reasons with those seeking care for non-functional reasons concluded that even if those seeking care for non-functional reasons exhibit a greater level of distress and problems relative to those seeking care for functional reasons, this still might not be clinically significant enough to warrant publicly funded plastic surgery [28]. Therefore, even if plastic surgery for functional conditions is not always more effective than that for non-functional conditions, the treatment effect might be more easily established from an objective standpoint. Once again, CJ1 does not necessarily align with PSE in all cases as to the effectiveness aspect.

#### Are functional conditions generally prioritized over non-functional conditions in the healthcare system?

Functional conditions are not necessarily prioritized over non-functional conditions in the healthcare system to the extent that there exist available and effective healthcare interventions. The dominant approach to classifying disabilities, the International Classification of Functioning, Disabilities, and Health covers both physiological symptoms, practical activities, and social aspects without giving precedence to any one of these aspects over the others [39]. In the healthcare system, there are examples where patients are provided with surgical interventions on psychosocial indications or due to experienced suffering. For example, requests for elective caesarean section due to a fear of childbirth are generally granted if the wish persists despite adequate therapy and psychiatric treatment, with the decision often motivated by the thought that not granting a caesarean section for these patients might lead to depression and post-traumatic symptoms [40].

However, there are examples elsewhere in the healthcare system where a desire for surgery based on anxiety and psychosocial conditions is denied. For example, women are generally not offered prophylactic mastectomies if they do not have a verified significant higher risk of breast cancer, such as a known mutation, even if they have a fear of breast cancer that affects their daily life [41]. In such cases, surgery is not considered the best treatment option, as prophylactic mastectomies, in cases where there is not an increased risk for malignancy, do not affect the incidence of cancer or life expectancy [42], despite the operation potentially decreasing patient anxiety. Similarly, removal of benign skin lesions is generally not offered [3], even if the patient is worried about malignancy. So, it seems that CJ1 might also violate PFE.

#### Conclusions concerning functional versus non-functional conditions

Despite the lack of consistency in how non-functional versus functional conditions are treated within the healthcare system, it is difficult to find a principled reason for an *absolute* priority of functional conditions taking precedence over non-functional conditions. Currently, both types of conditions are treated according to condition severity, alternative treatments, and treatment effectiveness. Generally, the concept of health implemented in the healthcare system do not support a sharp distinction between physical functionality and experienced suffering. Hence, CJ1 are not in equilibrium with our three principles PEN, PSE and PFE.

Nevertheless, functional conditions are somewhat easier to establish objectively, and the associated surgical intervention has a clear causal effect on treating the functional condition. It is also possible that a larger number of functional conditions exist with a corresponding high degree of severity relative to non-functional conditions.

### Non-functional appearance-related conditions

Having established the difficulty in normatively supporting a strict priority of functional over non-functional conditions (i.e. CJ1), we now focus on how to assess non-functional appearance-related conditions. Distinct from functional conditions, non-functional conditions are exclusively related to the experience and/or preference of the patient.

First, a short note on the distinction between subjective experiences of suffering and preferences related to plastic surgery and analysis of CJ2, i.e., that it should be enough to have a preference for plastic surgery from the patient to receive public funding. Someone might have a preference for plastic surgery without this being associated with suffering (e.g., they simply prefer to look different). If there are no other health conditions related to the condition (e.g., functional conditions), there will be no Z-gap (according to the definition of health above) and thus no healthcare need. Hence CJ2, does not align with PEN and our conclusion is therefore that patient preference is an insufficient criterion for publicly funded plastic surgery.

Establishment of a healthcare need requires at least some degree of suffering associated with the condition on which the patient is focused. The same physical feature can be associated with a suffering strong enough to result in a preference for its alteration in one person, whereas no such experience exists for another individual, resulting in no such preference. In this regard, many plastic surgery interventions are sensitive to preference variation (cf., assisted reproduction [43]). Therefore, the combination of having suffering and a preference for plastic surgery is a necessary criterion for publicly funded plastic surgery for non-functional conditions. We mean that suffering needs to be experienced in the sense that a person not *experiencing* suffering is not suffering [44]. However, suffering can be unwarranted, and we accept that suffering from different causes has different phenomenological qualities. For example, there could be a specific phenomenological quality to suffering related to appearance [45, 46].

A systematic review of physical and psychosocial outcomes after aesthetic surgery indicated that most patients undergoing aesthetic surgery have a lower pre-operative QoL (or greater suffering in our terminology) relative to controls, and that the procedure leads to a significant improvement that plateaus over time [34]. Similarly, another systematic review specifically focused on aesthetic rhinoplasty showed that studies suggest an improvement in QoL after the operation [35]. Nevertheless, these studies had methodological shortcomings, such as measurement imprecisions, variability in procedures and outcomes, and heterogenic and small study populations.

According to the strong emphasis on person-centered care, in which care should be adapted to the values, experience, and situation of the patient [17, 47], it could be argued that suffering caused by his/her appearance should warrant healthcare (i.e. CJ3). This provides prima facie support allowing the suffering to have sufficient weight in determining whether healthcare should be provided.

However, CJ3 is problematic from the perspective of PFE since different patients are differently equipped to report their suffering in a vivid and engaging way, thereby risking inequality. Moreover, it seems CJ3 is also problematic from the perspective of PSE. Even accepting that a problem exists, there might be better treatments for the experienced problem than surgery (e.g., psychotherapy, pharmacological treatment [38], or other psychosocial interventions [48]). Plastic surgery is generally not offered in the public healthcare system to address depression and psychosocial conditions due to bullying, although such patients assign their problems to an appearance feature, and studies show that many people who seek private plastic surgery as adults do so because they were bullied [49–51]. In assessing the size of the healthcare need, we need to assess the size of the gap between the current situation of the patient and the ideal situation (i.e., the problem of assessing the degree of suffering) in a systematic and consistent way to align with both PSE and PFE.

Examples of trusting self-reported experience to provide treatment include physical pain, which likely represents the best option for accepting self-reported experience at face value. However, even in these cases, providing a more potent treatment (e.g., morphine) with potential adverse side effects normally requires validation of the pain (e.g., in form of a reasonable biological rationale, such as suffering from cancer). PFE would then require us to do likewise when it comes to non-functional conditions and plastic surgery.

#### Conclusions concerning non-functional appearance conditions (at this point)

At this point in the analysis, we draw the following conclusions concerning conditions of a non-functional character requiring plastic surgery [i.e., conditions related to appearance or symptomatic conditions (not affecting function)]. To warrant public funding, the patient needs some form of suffering (and not only a preference for plastic surgery) that must be validated in some form by the healthcare system. This means that the healthcare system must have a reason to believe that the condition results in suffering. Ceteris paribus, the greater the suffering, the stronger the claim for public funding if the suffering is validated. However, at this point, what should guide the validation process remains an open question, given that a clear biological rationale might be lacking (cf., other somatic health conditions). In effect, neither CJ2 or CJ3 are in equilibrium with our three principles PEN, PSE, and PFE. Hence, we need to explore whether CJ4, where the suffering is validated in some form will fare better.

### Normality as validating non-functional conditions

We argued that for functional conditions, statistical normality is a valid criterion for determining whether a condition qualifies for public funding. Following this, we have a prima facie reason from PFE and will move directly from CJ4 to CJ5 (where the suffering is validated by reference to statistical normality) to explore whether CJ5 better aligns with our principles. In order to do so, we first need to distinguish between different uses of normality, where some are ethically unsuitable to be used as guides for reimbursement decisions, i.e. normative normality and normality as etiology. This will leave us with *statistical normality* as a potential guide also in relation to non-functional conditions.

#### Normative normality?

Normality is a complex, value-laden, and, to some extent, historically burdened concept that has been used to suppress groups that deviate from what is found as good or right in society [23]. Therefore, ‘normal’, on this understanding, is also considered ‘good’ (or ‘morally commendable’ or ‘the way things should be’), and the entire approach of norm criticism builds upon an idea concerning a problematic and restrictive value-laden characteristic of normality (e.g., in terms of hetero-normativity) [23]. The idea of what is normal appearance changes as a consequence of media and internet exposure to people who have had plastic surgery [7, 8], and patients pursuing private esthetic surgery now motivate their choice with a wish to be ‘normal’ [52]. The exposure to plastic surgery also increases patient expectations on what can be achieved by surgery [7, 8], which affects the perceived need of plastic surgery. Moreover, a positive experience of one plastic surgical procedure can trigger a perceived need for more plastic surgery to further improve body image satisfaction [53]. In healthcare, such a normatively laden view on normality should be avoided. Expressed in another way, normative normality is not consistent with accepting principles of equality and autonomy accepted at large in a democratic, liberal society and should therefore not be accepted within the healthcare system.

#### Normality as etiology

Another approach to normality that is potentially less problematic would be to evaluate the etiology of the condition. The following examples of etiologies appear relevant to public funding: malformations or tissue defects following cancer or trauma, but also conditions caused by medical treatment. When a patient has a crooked nose following a trauma, rhinoplasty is generally offered [54], whereas a congenitally crooked nose is considered a ‘normal anatomical variant’ and, therefore, not treated in the public healthcare system. When HIV treatment leads to lipodystrophy, with resulting suffering and social dysfunction, treatment with soft-tissue fillers, grafts, or lipofilling is usually offered [25, 55], whereas age-related facial grooves are not treated. Women with natural breast asymmetry need to meet rigorous criteria to be operated in the public sector [22, 56], whereas women who have had breast cancer are granted a contralateral procedure for symmetrization as a rule [57]. Moreover, in patients considered eligible for plastic surgery in the public healthcare system, corrections and revisions are generously performed upon patient request [58]. Patients with cleft lip and palate (CLP) are generally offered rhinoplasty to improve esthetics and ‘psychosocial rehabilitation’ [59], whereas unattractive noses without underlying malformation are not operated in the public healthcare system [60, 61]. What is the common denominator here? For the latter examples, if the healthcare system causes a condition (through treatment for some other condition), they have a specific responsibility to treat that condition. This does not cover the malformations resulting from cancer or trauma. A more general idea would be that conditions caused by some form of human choice or intervention would motivate funding; however, this neither covers malformations following cancer nor trauma. Although our goal here is not to explore all potential suggestions on the definitions of such etiologies, this discussion acknowledges the ambiguity in how this would be accomplished.

Why should the etiology of the condition matter? One idea is that a person adapts to his/her congenital appearance, whereas a *change* in appearance is more difficult to handle and entails more suffering. This would be a reason to operate a patient with a crooked nose following a trauma [54] but not the patient with a congenitally crooked nose. However, patients seeking plastic surgery for congenital conditions have not adapted to them but still suffer and are distressed (given the above requirement concerning objectively measurable suffering). If two different patients have the same appearance and similar suffering as a result, it is difficult to identify a convincing reason why the etiology of the condition would matter. In other words, normality as etiology does not align with PFE.

If a condition is the result of negligence on behalf of the healthcare professional(s), it can be argued that there is a moral responsibility to re-operate. However, if it is not the result of negligence but rather the expected outcome of a treatment indicated for the patient and which the patient had accepted based on information concerning expected iatrogenic conditions, it is difficult to assign a specific moral reason to prioritize such conditions before conditions with other etiologies. Consider the following example:

- Woman A suffering from breast cancer has undergone breast-conserving surgery, resulting in breast malformation after the wounds have healed. Another woman, B, is born with a somewhat worse form of breast malformation. Both are experiencing suffering an as a result of these malformations; however, the woman born with her condition has slightly more suffering due to the more pronounced malformation.

It is difficult to find a compelling reason for why woman A, having undergone breast cancer treatment, should be prioritized over woman B if B’s malformation is outside the normal range of variation. Following the reasoning in Beauchamp and Childress [15] on the non-maleficence principle, the harm caused by the breast-conserving surgery is a harm justified by the benefits from breast cancer treatment. From a priority setting perspective, this example could be described in a different manner:

- Woman A suffering from breast cancer experiences problematic symptoms and a high risk of premature death (a highly severe condition). The best and possibly curative treatment for woman A is breast-conserving surgery (together with other oncological treatments) along with the possible side effect of a breast malformation. With the breast-conserving surgery, woman A is brought from a very severe condition to a fairly mild one (i.e., the suffering resulting from breast malformation). Another woman, B, born with a discreet malformation also has a slightly more severe condition (a mild-to-moderate condition).

Described this way, it seems strange to claim that the healthcare system would have stronger reasons to spend more resources on woman A to improve her situation after having already applied resources for breast-conserving surgery than it would to spend resources on woman B with a somewhat worse condition. Hence, normality as etiology risks being in conflict with PSE.

It is difficult to identify convincing reasons why accounting for etiology would be ethically relevant when distinguishing how to publicly fund plastic surgery, unless we consider iatrogenic harm resulting from negligence. This does not imply that the described conditions do not warrant public funding, but rather that the etiology of these conditions does not provide an argument for this or apply a special standing to these conditions before other similar conditions with a different etiology. This since it does not align with PSE and PFE. A more promising argument would be that the conditions deviate from what is considered statistically normal.

#### Statistical normality

We then focus on *statistical normality* rather than collectively assumed conceptions of normality, societal normativity, or the etiology of the condition being more or less normal. Statistical normality should be grounded in research and not simply prejudice in order to align with PFE, since we generally require that healthcare is evidence-based. It is often presumed that esthetic conditions, such as facial disfigurements, are worse for females than for males [62], and that females are generally more distressed regarding their appearance [63]. However, recent studies reveal that many men experience body dissatisfaction [64, 65] and request a greater amount of esthetic surgery than previously [66]. Nevertheless, the myth that women suffer more appears to affect practice, as female CLP patients are offered significantly more surgical corrections than male CLP patients [67]. Another example of prejudice is that some conditions are considered more distressing for children than for adults [3, 68]. In practice, some procedures, such as otoplasty, are reimbursed only for children and adolescents and not for adults [3, 68], even though the procedure might reduce suffering in all age groups [69, 70].

There are great variations in specific or sets of physical features in a population. Moreover, some variations are more common than others in both appearance and functionality. From a principled perspective and accounting for a needs-based and publicly funded healthcare system with limited resources, it is reasonable to argue that the greater the deviation in the health of a patient relative to the majority of the population, the greater his/her healthcare need. If a patient has a common variation of health, and most patients are able to live with this variation without treatment – it is reasonable that such a variation results in lower claims to public resources. This is a standard approach within healthcare, in that it is accepted that patients have different blood pressure and cholesterol levels, knee mobility, and general symptoms within a ‘normal’ range of variation, and that such variation does not constitute a healthcare need that warrants treatment. Within the normal variety, there is a range of different risks of developing more serious conditions but also a range of levels of functionality or suffering. If a patient is within or above the normal range, they represent a positive outlier and do not qualify as having a healthcare need. This hence aligns with both PSE and PFE.

#### How does ‘normality’ relate to the concept of ‘healthcare need’ when considering non-functional conditions?

Similar to how statistical normality works in relation to a functional condition, it is also applicable for non-functional conditions. Normal variations could be ways to distinguish between non-functional conditions that warrant public funding and conditions that do not, despite the weak relationship between a specific physical feature and suffering, and the fact that a normal range is more difficult to establish.

As an example, all female breasts have a certain asymmetry. It is statistically rare within a perfectly symmetrical pair of breasts. Considering greater asymmetries (e.g., those due to underlying malformations, such as Poland’s syndrome [71]), these become more uncommon in the population. Given the need to prioritize scarce resources, consider the following situation:

- Patient A suffers from what she considers breast asymmetry, which is within a range common among women (40% of women have asymmetry within this range), most of whom do not experience suffering that results in their seeking healthcare.
- Patient B has the same level of suffering from what she considers breast asymmetry, which is extremely uncommon (< 0.5% of women have asymmetry within this range) and manifests as a large difference between the left and right breasts.

If both patients cannot receive publicly financed plastic surgery, how should such a choice be made? The two patients experience the same suffering from asymmetry; therefore, the amount of suffering per se is not grounds for distinguishing the conditions. The only difference is the statistical normality of the asymmetry, which is accounted for when assessing functional conditions. In these cases, functional disabilities appear to be a relevant parallel example. Even if two patients with a mobility condition experience the same degree of suffering, we would find greater reason to prioritize the patient with the greater mobility impairment. The strongest reason for this is equality. If people appear to be objectively on the same level, equality does not support using common resources to improve their situation further, especially if there are other people with worse conditions in the population. A reference level for equality is needed, and this is explicitly or implicitly represented by the general level of health of the normal population in terms of health-related QoL and life expectancy [72]. Another reason is that a publicly funded healthcare system needs to have democratic legitimacy, in the sense that the population should find the use of healthcare resources largely acceptable. It is unlikely that people would agree to spend resources on what is within a common variation as opposed to other less common maladies, especially if this common variation appears accepted by most people with the variation (i.e., Patient A). This is illustrated by the strong reactions from taxpayer alliances and the public when the matter is discussed [73, 74].

However, it can be argued that suffering rather than physical features should be taken into account. Consider the following two patients:

- Patient B suffers from what she considers to be breast asymmetry that is highly uncommon (< 0.5% of women have asymmetry within this range), with a large difference between the left and right breasts.
- Patient C reports a higher degree of suffering according to validated objective measurements from what she considers to be breast asymmetry, which is within a range common among women (40% of women have asymmetry within this range), most of whom do not experience suffering that results in their seeking healthcare.

In this situation, it is important to note that both patients are suffering from their condition and have a preference for plastic surgery; however, once again, we have to prioritize. There can be many explanations for a difference in suffering (e.g., people respond differently to what happens to them due to previous experiences and psychological robustness), as well as how people adapt to their present condition(s) [75].

Why should patient C not be prioritized in this case, given her greater suffering? It is not because her suffering is not real; this was validated as being greater than that of patient B. It is rather that her suffering does not appear reasonable according to the associated physical feature. There is a complex relationship between distress level and whether plastic surgery will improve the condition [36, 37]; therefore, distress level alone does not appear to be a sufficient measurement to establish whether publicly funded plastic surgery is indicated. In such a case, if both the normal range for the physical feature and the suffering are considered together, patient C should receive a lower priority than patient B.

#### Should society spend public resources on supporting alterations of physical features within the common range of the population?

Consider the following example:

- A man is suffering greatly, because he is unable to climb the Himalayas due to low oxygen consumption; however, his oxygen consumption is within the range of most people (perhaps a bit on the lower end) in his age category.

There is currently a treatment that would improve his oxygen consumption above the level of most people, thereby enabling him to climb the Himalayas. Should this be a priority for spending scarce public resources? The answer appears to clearly be ‘no’. Even if he is experiencing great but idiosyncratic suffering, it goes beyond what scarce resources should be used for as long as there are greater needs in the population. On the PFE, we should therefore not reason along these lines in plastic surgery.

One objection to this concerns person-centered care. What if this man used to be a mountaineer with a high oxygen-consumption rate that was lost due to a stroke? Should he not qualify for treatment to a greater degree than other patients with the same medical condition? This concept can be applied to plastic surgical procedures. For example, a woman diagnosed with bilateral breast cancer received an adequate breast reconstruction resulting in symmetrical normal-sized breasts; however, she previously had large breasts and wants to re-do the reconstruction to achieve larger-than-average breasts. Should society spend resources to for this breast reconstruction, despite her now having average-sized breasts? It is not unreasonable to adapt treatment to the individual circumstances and preference of a patient, and there are strong reasons to do this in circumstances when it can be done without extra expenditure. However, if such a situation requires more extensive resources, there are strong norms concerning equality against this, in that it is difficult to support taking resources from patients with greater needs (relative to a normal variation) in order to help people achieve a higher level of satisfaction than that within a normal range. Does it matter whether the procedure is related to an ability to work or a dependency on the extra resources to maintain career status? There might be exceptional cases, where people cannot continue to pursue a similar career without the extra resources.

Additionally, the concept of equity implies that it is unwarranted and inconsistent to add interventions not generally performed in publicly funded systems when a warranted intervention is performed in order to achieve a more ‘perfect’ result when this is done at an opportunity cost for other patients. This might be accomplished with minimal extra resources or where there is no real opportunity cost (i.e., the resource could not have been used for other interventions). For example, adding extra operating time when the patient is under anesthesia is a resource that cannot be used to operate another patient. The same argument can be used to ration the number of corrections and revisions offered to patients considered eligible for plastic surgery in the public healthcare system [58, 76]. For example, a woman receiving one reconstructed breast associated with cancer treatment needs to accept that the natural breast will likely result in breast asymmetry, despite a volume within the ‘normal’ range [77, 78]. This suggests that it is unreasonable to spend healthcare resources to achieve a perfect result in a patient with an acceptable breast-reconstruction result as it violates both PSE and PFE.

Similarly, equity also limits the influence of patient preferences when two treatments claim different resources. For example, in correcting breast asymmetry, should the patient be able to decide whether the smaller breast should be enlarged, even if it has a size within a normal range, or the bigger breast be reduced? Or even that both breasts should be changed? Such patient preferences must be balanced against other considerations, not least of which is resource constraints. Therefore, patient influence regarding exact treatment will depend on what is reasonable based on other competing conditions warranting public funding. Given this reasoning, the offer should remain consistent with what is offered to other patients and within the normal range of the population.

#### Treatment of incongruencies between the perceived identity and appearance

Would application of statistical normality as validation be at odds with the PEN, in the sense that acknowledged healthcare needs risk not receiving treatment using the concept of statistical normality? One group that does not fit into the discussion on ‘normality’ includes patients reporting an incongruence between their assigned sex and their experienced gender identity [79]. From a heteronormative perspective, these patients look perfectly ‘normal’, which is exactly the problem (normality norms cannot be applied to this patient group). For patients with a binary gender identity (having a male gender identity, despite being assigned a female sex and vice versa), there is a reference population that includes normal variations in the appearance of men and women in society. Therefore, patients with established gender incongruence and a binary identity could be offered plastic surgery to achieve an appearance within the normal variation of men and women in society. However, this might still imply limitations in terms of intervention relative to what the patient prefers.

A less clear-cut group includes patients with a non-binary gender identity (people identifying as neither male nor female, as both male and female, as different genders at different times, or as disputing the two-gender system) [80]. An example of a non-binary identity would be a biological woman who identifies as predominantly female but with strong aspects of the male gender [80]. The patient might want to keep her breasts but have smaller breasts than she has naturally, as she perceives big breasts as signaling a more female gender than her identity. Does such a patient applying an exclusive reference of self-identity have a larger claim to publicly funded breast reduction than a biological woman identifying entirely as female, but who wants smaller breasts for esthetic reasons/due to personal preferences? First, as in other cases, an objectively validated assessment of whether this causes the person suffering is needed. A personal preference for such a change is not enough in either case. Second, it has to be established whether the existing feature is within the common normal range. In the case of non-binary gender identity, this would imply that the gender type the individual wants to signal with a specific feature (i.e., a female gender with breasts) decides the normal range for this feature. Therefore, if the breast size is within the common normal range of the female gender, this will not give rise to claims to public funding; however, otherwise, it can.

#### Opening Pandora’s box?

Could the approach to this scenario open Pandora’s box for debate as to what should be publicly financed? Many patients seek private plastic surgery due to an ethnically typical nose [60, 61] or eyes [81]. However, if this ‘ethnic’ appearance is commonly represented in the population at large, it will not provide grounds for public funding. In an ethnically diverse society, where most ‘ethnic’ appearances are fairly commonly represented, most patients experiencing this condition would likely not get publicly funded plastic surgery based on application of the statistical normality criterion. Moreover, patients often approach plastic surgery with requests to look younger [82]. In both cases, the patient could arguably have an incongruence between perceived identity and appearance features, such as ethnicity and age. If gender incongruence is accepted as a healthcare need, why not other identity incongruences, such as age? Gender incongruence is an established diagnosis with professional treatment guidelines [83], and as long as age incongruence or other such incongruencies are not generally accepted in healthcare, they cannot be considered relevant healthcare needs with potential claims on healthcare resources, i.e. they would not align with PEN. Nevertheless, there has been a shift over time regarding perceptions of the differences between which conditions constitute suffering and what is viewed as a healthcare condition that should be treated within the public healthcare system. Hence, over time we might have reason to re-evalutate this.

In conclusion, CJ5: “If patients have validated suffering that can be reduced with plastic surgery and their condition is outside the range of what is statistically normal, it should be publicly funded”, would seem to have some initial plausibility to constitute a hypothetical equilibrium for what should guide public funding for plastic surgery. Of course, with the proviso that it needs further testing against a broader set of principles and considerations.

### Study limitations and future perspectives

This is the first ethical analysis performed on general principles of prioritizing plastic surgery; therefore, there is a need for further discussion of the principles and conclusions presented here. Even if the methodology of reflective equilibrium does not strive for agreement between different stakeholders per se*,* a part of the methodology is that the analysis and argumentation should be critically reviewed and part of a developed academic discussion before being accepted. This critical review implies reviewing the arguments and judgements for consistency and relevance. To achieve trustworthiness of the analysis, it was performed by a medical ethicist in collaboration with a plastic surgeon, and have greatly benefited from comments of a number of reviewers. Still, to further strengthen the trustworthiness of the argument, critical discussion and analysis of the suggestions we present in this article is necessary.

Moreover, in this article we have only strived for a narrow reflective equilibrium, looking almost exclusively at central normative principles guiding distributive justice in healthcare. On a wider reflective equilibrium we need to broaden the scope. For example, look at psychological patterns and attitudes, feasibility of applying our approach in terms of patient, professional and public acceptance and legitimacy as well as whether consistent application of the concept of statistical normality within the field of appearance would be possible. A further complication with reflective equilibrium is what we should consider as “resting points”, i.e. more established views that we should be more resistant towards revising, be it considered judgements or principles. In this article we have taken our three principles for granted; however, they could obviously be revised and refined in different ways, enabling equilibrium with other sets of considered judgements. To our defence, the principles we have used are well-established and widely accepted in many healthcare jurisdiction of welfare type, with far-ranging consequences to abandon^1^.

The focus on exploring general principles related to public funding of plastic surgery implies that detailed arguments need to be developed before they can be used to guide reimbursement of plastic surgery in the public healthcare system. Such developments should also include a more systematic testing of concrete cases against the principles and empirical studies on normality and appearance. Hence, in order to support the analysis in this article, further empirical studies are needed.

## Conclusions

In exploring functional versus non-function conditions, we demonstrated the difficulty in finding a principled reason for an *absolute* priority of functional conditions over non-functional conditions. Nevertheless, functional conditions remain, to some extent, easier to establish objectively, and the surgical intervention has a clear causal effect on their treatment.

Considering non-functional conditions requiring plastic surgery (i.e., conditions largely related to appearance), we concluded that the patient needs validated suffering according to the healthcare system.

This validation is required for both functional and non-functional conditions. Functional conditions were validated by distinguishing between statistically normal and abnormal functions. Similarly, for non-functional conditions, we determined statistical normality as a potentional validation concept for distinguishing between what should and should not be publicly funded. However, we acknowledge that such a concept needs further development.

## Data Availability

Not applicable.

## Abbreviations

CLP: Cleft lip and palate
ICF: The International Classification of Functioning, Disabilities and Health
HIV: Human Immunodeficiency Virus
QoL: Quality of life

## Footnotes

1 From a meta-ethical perspective, reflective equilibrium could be criticized both on ontological grounds, for assuming an unwarranted constructivist view on ethics, and on epistemological grounds for being vague about what constitutes coherence. However, it would go to far to venture into a discussion of these criticisms in this article.
